# Estimating the Potential Impact of the 2024 UK Salt Reduction Targets on Cardiovascular Health Outcomes and Health Care Costs in Adults: A Modeling Study

**DOI:** 10.1161/HYPERTENSIONAHA.125.25159

**Published:** 2026-01-26

**Authors:** Lauren Bandy, Ben Amies-Cull, Madison Luick, Linda J. Cobiac, Susan A. Jebb, Peter Scarborough

**Affiliations:** 1 Nuffield Department of Primary Care Health Sciences, University of Oxford, United Kingdom (L.B., B.A.-C., S.A.J., P.S.).; 2 Oxford Population Health, University of Oxford, United Kingdom (M.L.).; 3 School of Medicine and Dentistry, Griffith University, Brisbane, Queensland, Australia (L.J.C.).

**Keywords:** adult, cardiovascular disease, health care costs, nutrition surveys, policy

## Abstract

**BACKGROUND:**

Excessive sodium intake is responsible for 3 million deaths a year globally. The UK is one of 64 countries to have a salt reduction program to help reduce the population’s sodium intake. It is a voluntary scheme with 108 category-specific salt content targets for the grocery and out-of-home sectors. This study aimed to estimate the potential impact of the 2024 targets on cardiovascular outcomes and health care costs for UK adults.

**METHODS:**

Long-term health modeling was based on the adult population in England. Changes in salt intake (g/d), whether the targets were met, were estimated using consumption data from the National Diet and Nutrition Survey 2018/19. Impact on ischemic heart disease and stroke, quality-adjusted life years, and health care costs were estimated using PRIMEtime, a proportional multistate life table model.

**RESULTS:**

If the salt reduction targets set for 2024 had been met, then salt intake would have reduced from 6.06 g/d (95% CI, 5.18–6.31) to 4.94 g/d (4.73–5.15), a reduction of 1.12 g/d (1.05–1.20). This would lead to 103 000 (UI, 41 000–161 000) fewer cases of ischemic heart disease and 25 000 (10 000–39 000) fewer cases of stroke over 20 years. A modeled 243 000 (94 000–383 000) quality-adjusted life years would be saved with a net saving of £1.00 billion (£0.35–1.73 billion) to the National Health Service over the remaining lifetime of the adult population.

**CONCLUSIONS:**

Reformulation of products to meet the targets could result in substantial reductions in cardiovascular disease without changes in dietary behaviors. Policymakers should consider options to strengthen salt reduction policies, including effective systems for monitoring and enforcement.

**NOVELTY AND RELEVANCE**

**What Is New?**

This is the first study to model the impact the UK Government’s category-specific salt reduction targets could have on the population’s cardiovascular health should they be achieved in full by the food industry

**What Is Relevant?**

Our results show that, should the targets be met, there would be reductions in the population’s salt intake and subsequent reductions in ischemic heart disease and stroke.
Salt reduction targets are an important health policy tool that can help prevent diet-related ill health at the population level and lead to important reductions in health care costs.

**Clinical/Pathophysiological Implications**

Reducing the population’s sodium intake can lead to important reductions in blood pressure and reduce the risk of cardiovascular disease. Reducing dietary sodium should be discussed by clinicians with patients with hypertension.

Excess dietary salt is responsible for 3 million deaths a year globally.^1^ There is strong evidence that reducing dietary sodium leads to dose-dependent declines in both systolic and diastolic blood pressure,^2–4^ which are key risk factors for cardiovascular diseases.^5,6^ The World Health Organization recommends that salt intake should not exceed 5 g/d and that salt reduction is one of the most cost-effective measures a country can take to improve population health.^7^ The UK government’s salt reduction program first began in 2003 and consisted of a set of voluntary targets as well as product labeling, a public and political awareness campaign, and monitoring.^8^ Since then, the campaigning element of the salt reduction program has diminished, but the Government has published a series of updated targets for industry in 2009, 2011, 2014, 2017, and mostly recently in 2020 to be met by 2024.^9^ Sales-weighted mean salt content and maximum salt content targets were set for 84 grocery food categories, such as bread, cheeses, meats and snacks, and, for the first time, 24 food categories consumed out of the home, including chips, burgers, curries, and pizza.^9^ The aim of these targets has been to encourage manufacturers to gradually reduce the salt content of everyday foods so that consumers do not notice a change in taste; therefore, reducing the impact on consumer preference or product sales.

Previous studies have reported that initially, these targets were successful at reducing sodium consumption. Population salt intakes in England fell by 15%, from 9.5 g/d in 2003 to 8.1 g/d in 2011.^10^ Over the same time period, a fall in the population’s blood pressure of 3.0/1.4 mm Hg (diastolic/ systolic) was observed, contributing to an estimated 42% and 40% reduction in stroke and ischemic heart disease (IHD) mortality, respectively.^10^

More recently, however, there have been concerns that progress in reducing dietary sodium intake has stalled.^11^ Dietary survey data shows that the UK population’s salt intake has begun to increase, from 8.1 g/d in 2011 to 8.4 g/d in 2017 to 2018.^12^ Between 2015 and 2020, the mean salt content of grocery foods in the UK fell by just 0.05/100 g, with little to no change in leading grocery categories, such as bread, cheese, and ready meals—lack of engagement by the food industry with the salt reduction program being a contributing reason.^13^ A 2020 report on the food industry’s progress towards the 2017 targets by Public Health England showed mixed results, with only half of the grocery targets being met.^14^ With a new government and salt targets due for review, it is pertinent to examine the potential benefits of salt reduction for population health.

This study aimed to estimate the potential impact the 2020 UK salt reduction targets would have on average UK salt intakes if they had been met in full by the food industry by 2024, and to estimate their potential impacts on cardiovascular outcomes and health care costs at the population level.

## Methods

### Data Availability

The National Diet and Nutrition Survey (NDNS) data 2019 that was used in this study is available through the UK Data Service at the following link: https://beta.ukdataservice.ac.uk/datacatalogue/series/series?id=2000033. Other supporting data and materials are available within the article, and it is in the Supplemental Material.

We modeled a scenario estimating the potential changes in population salt intake, IHD, stroke, and health care costs that could be achieved by the government’s 2020 to 2024 salt reduction target program for foods consumed both in and out of the home in the United Kingdom. Ethics committee approval was not required.

### Estimating Changes in Salt Consumption

The NDNS is a rolling annual cross-sectional survey that collects data on food consumption and nutrient intakes of a representative sample of ≈1000 individuals (around 500 children and 500 adults) using repeated food diaries for a 3 to 4 day period to account for day-to-day variation.^15^ The NDNS data is weighted to adjust for differential selection probabilities of households and individuals (age, sex, and region—based on mid-year population estimates for 2017^16^), and nonresponse rates to questionnaires, nurse’s visits, and blood sample collection.^17^ Data are published by the UK Government and were accessed through the UK Data Service.^18^

Individual-level microdata on the volume of food products consumed (g/d) and their corresponding salt content data (g/100 g) were taken from NDNS year 11 (2018–2019). Each food product was matched to one of the 108 categories covered in the salt reduction program, based on its category name and product description. The salt target category names and the number of foods from NDNS matched to each category are given in Table S1. NDNS survey weighting values were applied to increase the representativeness of the sample to the UK population. The baseline daily salt intakes (g/d) for adults by sex were calculated by summing salt intake across all food categories for preprepared and packaged foods, including those purchased from takeaways (ie, foods that fell outside the salt reduction target categories, or were described as homemade, were not included in baseline salt intakes in this paper).

The salt content (g/100 g) of each food was then repopulated with the category-specific target values given in the salt reduction program. Foods that were described as takeaway were categorized according to the out-of-home sector targets as given in the salt reduction program. The targets for the out-of-home sector are given per serving size, not per 100 g; therefore, it was assumed the reported total grams consumed variable in NDNS was equivalent to serving size. The scenario daily salt intake (g/d) for each adult was calculated by summing salt intake across all recorded food categories using these re-populated target salt values for foods.

We corrected for underreporting in the dietary recall questionnaire of the NDNS by assuming the pattern of underreporting in the dietary recall questionnaire was equal across all foods. We did not adjust for the salt target categories that could not be matched in the NDNS data. A correction factor was calculated as the ratio between the published mean estimated salt intake from the NDNS England Sodium Survey 2018/19^12^ and baseline mean salt intakes estimated in this study, by age group (19–34, 35–49, and 50–64 years) and sex, as published in the National Sodium Survey. The sodium survey is based on urinary sodium, so the ratio represents the proportional difference between what is reported in the dietary survey (whether from food products or added salt) and what can be inferred they are consuming (whether from food products or added salt). The estimates of salt consumption at baseline and scenario were multiplied by these factors, and differences were calculated between the 2 to capture the change in salt consumption related to the targets, assuming that people are under-reporting consumption of target and nontarget food categories equally. We conducted a sensitivity analysis where there was no adjustment for under-reporting of salt consumption.

### Estimating Impacts on Disease Burden and Health Care Costs

The 2020 population of the UK was used from the Office for National Statistics, baseline disease epidemiology from the Global Burden of Disease 2019,^19^ with secondary modeling using DISMOD-II,^20^ and blood pressure-disease relationships were quantified using Relative Risks from a meta-analysis of prospective cohort studies.^21^ The impact of the scenario changes to mean salt intakes on the disease burden of IHD and ischemic stroke was modeled using the PRIMEtime model. This is a population model using a proportional multistate lifetable model structure, which has been described fully elsewhere, including all its epidemiological parameters.^22,23^ A brief description of how it operates is as follows. The basic principle is of a 3-state lifetable where a population is categorized as no disease, disease, or dead from disease, with incidence rate acting as the first state transition parameter and the case fatality rate as the second, assuming remission from the disease outcomes equals zero. First, the risk module quantifies the impact of the change in risk factor on the incidence of diseases. This is done by calculating the population impact fraction for each disease outcome, which represents the proportional difference in disease incidence related to a change in a related risk factor, calculated as population impact fraction=(∑_*i=n*_, *p*_i__*_ *RR*_*i*_−∑_*i=n*_, *p’*_i__*_ *RR*_*i*_)/∑_*i=n*_, *p*_i__*_ *RR*_*i*_, where RR is the relative risk of a risk factor exposure category, p is the baseline prevalence of that risk factor exposure level, p’ is the equivalent scenario prevalence, and i represents each category of risk factor exposure and n is the number of categories. It, therefore, represents the proportional change in the sum of products of categories of risk factor exposure level with those categories’ relative risks on disease outcome. Here, the relationship between salt and blood pressure (BP) is linear and the relationship between BP and disease outcomes is nonlinear (parabolic with a theoretical minimum risk of 115 mm Hg), using a conversion factor of 5.80 mm Hg (millimeters of mercury; 2.45–9.15 mm Hg) per 100 millimoles of sodium (equating to 2.30 g of sodium or 5.88 g of salt)^4^ and calculating the population impact fractions for IHD and stroke using relative risks on BP. A change to disease incidence due to the intervention is calculated as I′ =I*(1−population impact fraction), where I is the baseline incidence rate and I′ is the scenario incidence. Increased disease burden is modeled as a downstream consequence is this increase in incidence. Uncertainty intervals for scenario changes to BP are calculated using 10 000 iterations of Monte Carlo analysis. Second, the disease module separately quantifies the impact of the change in disease incidence on disease burden over time, and finally, the life table module compiles these disease burden impacts into a total population burden. The population is structured by age and sex, and is simulated to age across the chosen time horizon simultaneously with baseline and scenario disease incidence, allowing the difference between the 2 to be calculated.

The reduction in salt intake was implemented linearly over the 4 years, and the baseline comparator was for no change to mean salt intakes. Impact on life expectancy was calculated from the change to all-cause mortality rates, quality-adjusted life year (QALYs) were calculated using disease utility decrements from a UK study,^24^ and unit health care costs were calculated as previously described for PRIMEtime.^22,23,25^ Modeled impacts on QALYs and health care costs were estimated over the remaining lifetime of the population, and impacts on disease cases were estimated over 30 years, each using closed cohorts. Costs of future unrelated diseases were not included, and discount rates of 3.5% were used for disease burden and health care costs, in line with guidance from the National Institute for Health and Care Excellence (NICE).^26,27^ A time lag of 5 years was set between changing BP and the increase in disease incidence rates.^23^ Disease incidence and case fatality trends were incorporated for 10 years by projecting forward the past 10 years from the Global Burden of Disease.^19^ Population BP was assumed not to change by other means over time, as a change to population-level systolic BP is likely to be largely a consequence of interventions such as the 1 described here, so cannot be assumed to be happening in the baseline simulation. Monte Carlo analysis included uncertainty on BP-disease Relative Risks, the salt-BP relationship, baseline state utilities, disease utility decrements, health care costs, and the intervention effect (with additional parameter values included in the Supplemental Material).

**Figure 1.**
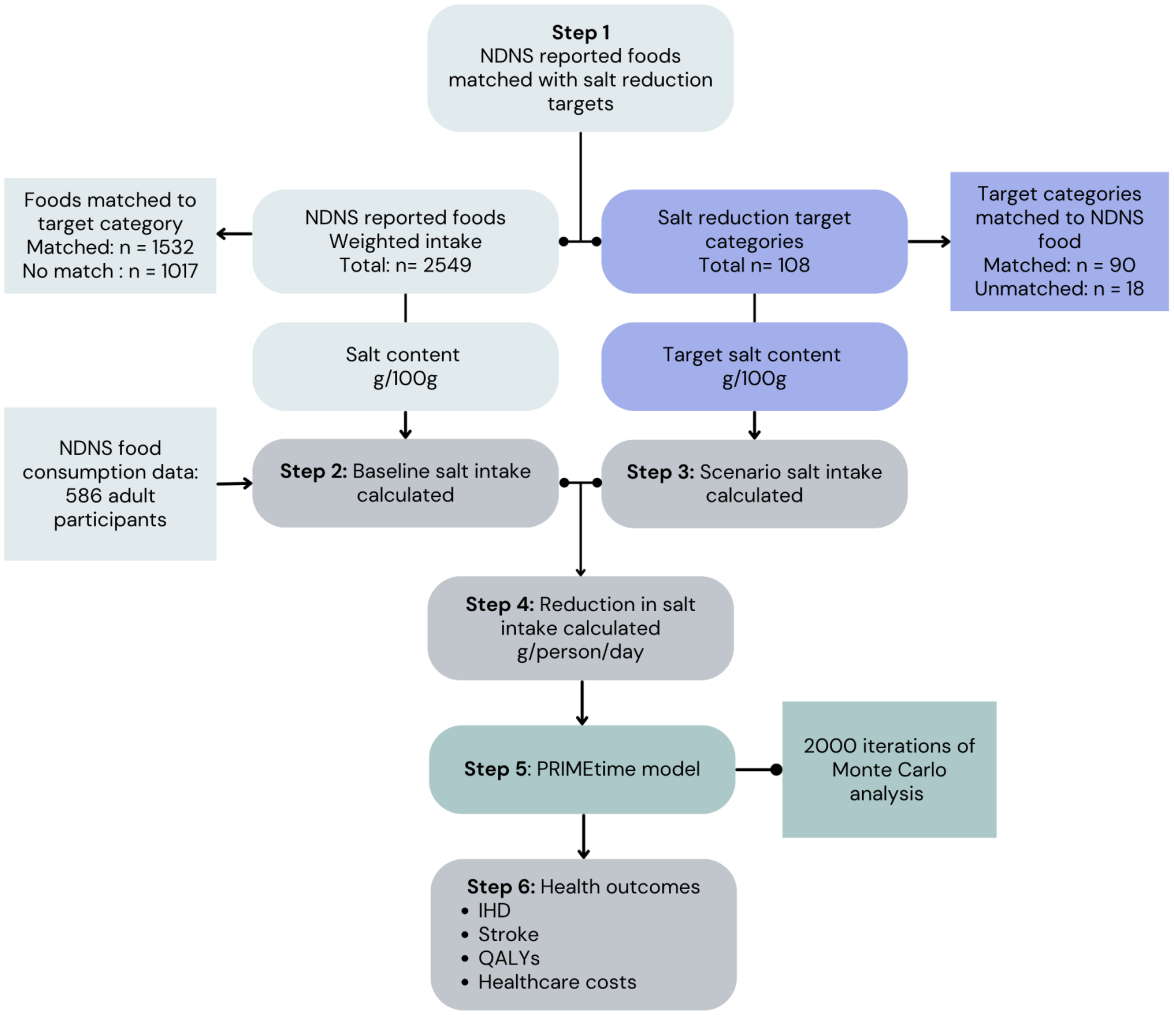
**Summary of methods.** IHD indicates ischemic heart disease; NDNS, National Diet and Nutrition Survey; and QALY, quality adjusted life year.

A flow chart summarizing the methods is given below (Figure 1).

### Key Assumptions

The scenario was modeled assuming that the program had no impact on consumer behavior. Specifically, it assumes that people eat the same food products, in the same quantities, and do not add any additional table salt, so they consume less salt because of reformulation. It assumes industry is effective at reducing salt consumption across the range of targets.

The analysis took 2 separate steps: a static microsimulation of potential impact on salt reduction and a population modeling simulation for the health and health care cost impacts of these reductions in salt consumption. The Proportional Multistate Lifetable Model structure makes key assumptions in causality of salt onto BP and BP onto IHD and stroke outcomes. Diseases are treated as independent end points with an average severity applied for calculating QALYs, and the prevention of a case is assumed to linearly save health care resources. In the context of a model designed for use on disease prevention scenarios, the lack of ability to capture change in the severity of cases is unlikely to be relevant.

### Sensitivity Analyses

Three sensitivity analyses were included using point estimates and 500 iterations of Monte Carlo analysis.

1. A reduction in the effect to represent unintended public and industry response, such as failure to achieve reformulation or the public adding more table salt, was modeled at 20% and 40% less than the main analysis across included products.
2. Alternate discount rates of 1.5% and 0% were applied to modeled outcomes as point estimates (consistent with the NICE’s recommended discounting comparators).
3. Baseline salt consumption was reduced (and therefore also the effect size of the intervention) due to no adjustment being made for under-reporting of salt consumption.

## Results

### Sample

A total of 586 adult participants aged 18 years and over in the NDNS were included. This sample was weighted to be representative of the UK population based on mid-year estimates for 2017.^16^ Two thousand five hundred forty-nine unique foods were reported to be consumed by this sample, of which 1532 were matched to a salt reduction target category (Table S1).

### Allocating Products to Salt Reduction Categories

It was possible to match 75 out of the 84 (89%) grocery food targets and 8 out of 24 (33%) of the out-of-home sector targets to their related foods.

### Salt Consumption

**Figure 2.**
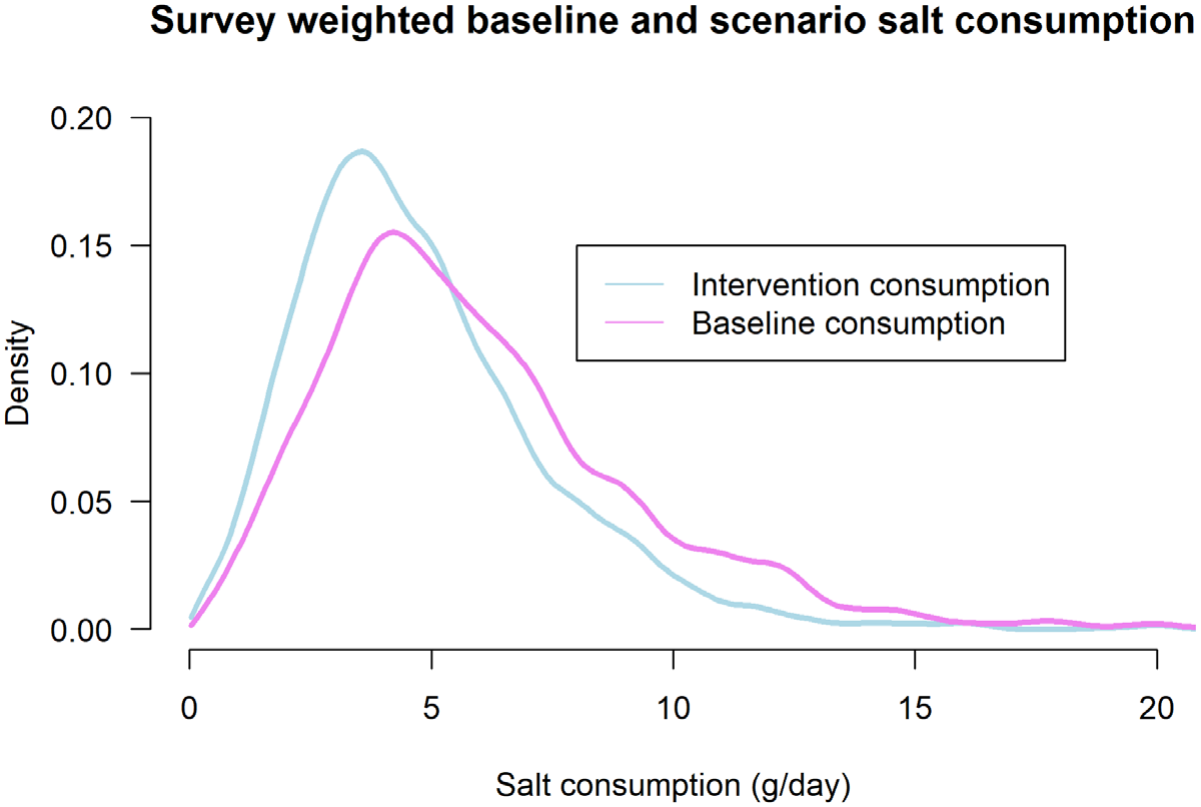
Survey-weighted distribution of salt intake at baseline and intervention.

If the government’s category-specific salt reduction targets set for 2024 were achieved in full by both the grocery and out of home sectors, it is estimated that the adult population would see their salt intake reduce from 6.06 g/d (95% CI, 5.18–6.31) to 4.94 g/d (95% CI, 4.73–5.15 g), a reduction of 1.12 g over 4 years (95% CI, 1.05–1.20 g) equivalent to 17.5% (Table 1). The reduction would be greater for males (1.34g [95% CI, 1.22–1.47 g) than for females (0.93 g [0.85–1.02 g]). The distribution of salt consumption in the adult population at both baseline and intervention is shown in Figure 2.

**Table 1.**
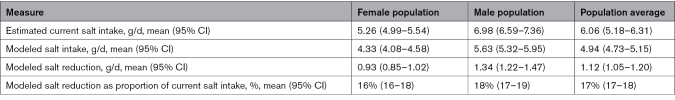
Salt Intake for Adult Population Aged 19 to 64 Years, Current, and Modeled Intervention (Should Salt Reduction Targets be Achieved) With 95% CI

### Disease Burden and Health Care Cost Savings

Reduction in systolic BP was calculated as 0.919 mm Hg (0.392–1.46 mm Hg) for females and 1.32 mm Hg (0.561–2.11 mm Hg) for males. In the first 20 years, this could avert 103 000 (95% UI, 41 000–161 000) cases of IHD and 25 000 (95% UI, 10 000–39 000) of ischemic stroke across the population (full breakdown in Table 2 below). Over the lifetime of the population, accounting for the potential for poorer health in later life, it would save 243 000 IHD—and stroke-related QALYs overall—77 000 for women (95% UI, 30 000–121 000) and 166 000 for men (95% UI, 64 000–263 000). The health care cost savings to the National Health Service are estimated to be £1.0 billion (95% UI, £351 m to £1.73 billion) over the population’s lifetime. Life expectancy changes were estimated to be 0.0174 years (0.00718–0.0290) for females and 0.0418 years (0.0175–0.0683) for males, or 6.35 days and 15.3 days, respectively.

**Table 2.**
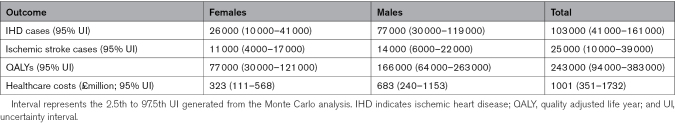
Estimated Impact of 2024 Salt Reformulation Targets Being Met on Reduction in QALYs, Disease Burden, and Health Care Costs

### Sensitivity Analyses

The results tables of the sensitivity analyses are presented in the Supplemental Material. The first sensitivity analysis shows that even if manufacturers reduced salt intake but missed the category targets by 20% or 40%, the reduction in the population’s salt intake would be less, but there would still be significant savings to QALYs and health care costs for males and females (Table S6). These show reductions in potential effects approximately proportional to any shortfall in the desired impact of the targets on salt reduction, and that future discounted benefits are considerable, so need accounting for in long-term decision-making. Our sensitivity analysis, where we did not adjust for under-reporting of salt consumption, produced a baseline salt consumption of 5.21 g/d in our sample (compared with 6.06 g/d in our primary analysis). In this sensitivity analysis, the percentage reduction in salt consumption remained the same, but due to the smaller baseline, the absolute amount of salt reduction was reduced by about 13%. Consequently, our estimates of the health impact of the intervention were also reduced by ≈13% (Table S8).

## Discussion

This study shows that if the UK Government’s salt reduction targets for 2024 were met in full, it could lead to a 17.5% reduction in the salt intake of the adult population. This would prevent an estimated 103 000 cases of IHD and 25 000 ischemic strokes over 20 years, leading to a saving of 243 000 QALYs and saving the National Health Service £1.00 billion over the population’s lifetime. Should the program not meet its targets, for example, a 20% or 40% shortfall, health benefits may be lost approximately linearly with these shortfalls. This study demonstrates that if implemented as intended, the UK salt reduction program could have significant benefits for the population’s health. These effects are likely to be underestimated here, given that limitations in both the NDNS data and salt reduction target category definitions mean that only 84 of the 108 target categories could be matched to foods reported in NDNS. The average reduction of systolic BP in our study was modest, at 0.919 mm Hg and 1.32 mm Hg for females and males, respectively.^28^ This compares with an average treatment effect of 5.1 mm Hg for a first BP medication, though here the intervention affects many more people, over the entire life-course, and avoids up-front costs to the National Health Service. Likewise, though life expectancy impacts are modest, it is important to note that these benefits are averaged across many more people than the change to disease cases, so smaller numbers of people may have comparatively large benefits to disease burden.

### Comparison to Literature

This study is the first to estimate the potential public health impact of the UK Government’s current salt reduction policy and adds to the existing modeling evidence that population-level reductions in salt intake have the potential to lead to improvements in health outcomes. A previous study has estimated the cases of premature IHD and stroke averted in the UK based on (1) if the salt intake reduction of 1.0 g/d between 2000 and 2018 was maintained to 2050 and (2) projected gains of reaching the World Health Organization guideline of 5 g/d per adult by 2030.^29^ The study, using the same multi-state life table model structure (PRIMEtime) as in our study, found that 83 140 cases of IHD and 110 730 strokes could be prevented by 2050 if previous reductions in salt intake were maintained to 2050, equivalent to £1260 million in health care savings.^29^ The salt reduction modeled in our study was slightly greater, particularly for males, and while the overall effects are comparable for IHD cases, they are somewhat lower for stroke cases.

Other studies have modeled the health and cost benefits of achieving a general reduction in the population’s salt intake (eg, 3 g/d reduction or 25% reduction), including in European countries,^30^ Australia,^31^ India,^32^ and United States,^33^ but there are fewer studies that look at the impact of sodium reformulation targets specifically.

A modeling study conducted in New Zealand found that implementing mandatory sodium targets for packaged foods and fast foods sold in restaurants would lead to a 35% reduction in the population’s salt intake and result in 235 000 QALYs gained over the cohort’s lifetime.^34^ Two studies conducted in Australia that used similar methods using sales-weighted salt content data for food categories from commercial home-scan data to estimate the population’s sodium intake from packaged food before and after Australia’s national category-specific salt reduction targets had been met.^35,36^ If full compliance were achieved, 1 study estimated it could prevent 1920 (95% UI, 1274–2600) cases of CVD, chronic kidney disease, and stomach cancer per year,^36^ with the other estimating 12 722 (7755–18 653) cases of CVD over 10 years.^35^ Another Australian study with similar methods compared the World Health Organization’s category-specific targets to the Australian national policy, and it was estimated that if the World Health Organization’s targets were achieved in full, then it could lead to 7000 fewer cases of CVD, chronic kidney disease, and cancer a year.^37^ Overall, the findings presented in this study match the conclusions already existing in the literature—that category-specific reformulation targets could have substantial public health benefits, should they be complied with.

Previous PRIMEtime modeling explored the potential impacts of the UK Government sugar reduction strategy on health, with analogous voluntary reformulation targets. At 1.5% discount rates, this found the strategy could save 839 000 QALYs and £2.71 bn over the lifetime.^38^ This study estimates potential benefits of less than half that for the sugar reduction strategy, though that paper highlighted that not all these benefits were likely to be seen as the government was aiming to achieve sugar reduction through weak assumptions, such as consumers choosing healthier choices. Here, the opposite issue may be present—the inability to allocate all relevant products to their categories means some potential benefits of the program may not be captured here.

### Limitations

The NDNS provides evidence on the UK population’s diet, but has several limitations. The main one is that the nutrient database linked to NDNS foods is not updated regularly. This means that the salt content of the foods we have used to estimate baseline salt intake is not accurate as of 2024, with the salt content of some food categories based on generic products rather than products found on the market.^39,40^ The results presented here are therefore a hypothetical exercise in what could have been achieved if the targets were met. It is possible that for some categories, we are accounting for reformulation that may have already occurred, and the health benefits are already being seen. However, evidence shows that between 2015 and 2020, there was little change in the mean salt content of grocery foods over time, at −0.05/100 g or −4%. Given this previous trend, it seems unlikely that these targets are going to have been met in full by the industry. An up-to-date survey on the salt content of foods in the UK is recommended for future research.

NDNS data has other limitations. First, the majority of participants are white, with ethnic minority households under-represented.^41^ Second, the reliance on self-reported data means that consumption is often under-reported. We adjusted on the basis that the under-reporting of salt intake was proportional to urinary sodium, but it is important to highlight that the urinary sodium survey also suffers from a small sample size, selection bias, and incomplete sample collection. Also, while this adjustment will account for under-reporting of processed and preprepared foods, it will also account for salt added at the table and during cooking, which is not covered by the targets. The latter reason may mean that our adjustment is not conservative, which is why we included a sensitivity analysis that removes the adjustment. NDNS identifies foods that are purchased out of the home, and they are coded as takeaway. However, there is significant underreporting of foods purchased as takeaway, with only 8 of the 24 targets for the out-of-home sector matched to NDNS.

That some food categories could not be matched to their target products conferred a conservative bias on the estimate of salt reduction under these targets. It is not possible to meaningfully estimate to this bias affects the estimates, as the ambition of the targets and the quantities consumed of each product both vary greatly.

The scenario modeling relies on observational studies to link BP to disease outcomes,^21^ so unobserved confounding of effect sizes is possible. The PRIMEtime modeling structure assumes the risks of disease outcomes are independent, which may confer a conservative bias on results due to not accounting for superadded disutility and treatment costs associated with multimorbidity. In addition, only IHD and stroke are modeled here, while salt consumption is also potentially linked with diseases such as chronic kidney disease, gastric cancer, and dementia, conferring further conservative bias. We assume a flat linear salt-BP relationship, though there are possibly subgroup variations in this effect. It is unlikely that this assumption confers an overall bias due to the intervention affecting the whole population and lifetime.

### Policy Implications

These results demonstrate the public health benefits that might be achieved if the salt reduction program were successful. This study shows that the mean pace of salt reduction attributable to the salt reduction targets could be 0.28 g/d per year over 4 years, and compares favorably to the previous success seen between 2003 and 2007 when the salt reduction program was originally introduced, which achieved a rate of reduction of 0.175 g/d per year.^42^

This analysis was limited by related issues in that it was not possible to allocate many products to target categories, indicating potential weakness in monitoring progress towards the targets. Some target categories are overly specific; for example, the target for blue cheese (grocery category, 4.4) is only relevant to products manufactured in the United Kingdom, which means many products are exempt from the target, but also that origin data is also needed for monitoring industry compliance. The targets for battered or breaded chicken purchased out of the home are split based on energy content: under 200 kcal per serving, 200 to 400 kcal, and over 400 kcal, making the data required to monitor them even more complex than the per 100g targets set for grocery foods.

If policymakers elect to stick with the voluntary program, then more meaningful progress might be achieved by investing in better monitoring and more regular reporting. For example, including progress towards the salt targets as a metric that businesses should declare as part of a mandatory reporting system^43^ might lead to greater public and political scrutiny, and therefore, more meaningful changes by manufacturers.

An alternative option is to make the targets mandatory, although enforcement of a mandatory scheme—for example, implementing a system to monitor and remove products from the shelves or issue fines for noncompliance—is likely to come at a high economic cost. Of the 62 countries that have salt reduction strategies worldwide, mandatory targets are in place in 9, with a further 6 having a combination of mandatory and voluntary schemes.^44^ Only 3 of these—Argentina, Paraguay, and South Africa—have evaluated their mandatory schemes using labeling surveys led by independent academic groups.^44^ South Africa, for example, has implemented a 2-phase mandatory salt target program for 13 food categories that contribute the most to sodium intake, with interim targets introduced in June 2016, followed by stricter targets in June 2019.^45^

A 2021 survey found that 75% of products overall had a sodium content at or below the targets according to their labels, although there was great heterogeneity between categories.^46^ The same survey also found that for certain categories, including bread and processed meat, the sodium content of products estimated from laboratory analysis was significantly higher than the values reported on the labels, which could overestimate compliance rates.^46^ Given the variation in salt strategies implemented and evaluation methods used across countries, it is hard to conclude whether mandatory targets are more or less successful than voluntary targets in achieving both compliance and population-level health benefits.^46^

### Conclusions

This study found that if the 2024 salt reduction targets had been achieved in full by the food industry, a major population-level reduction in sodium consumption and its associated disease burden would be predicted, with an estimated average of 17.5% less salt being consumed per person per day. This could be associated with 103 000 cases of IHD and 25 000 strokes in 20 years, accumulating to 243 000 QALYs and £1.0 bn in health care cost savings over the lifetime of today’s adult population. Given these benefits, this study highlights the importance of prioritizing salt reduction as part of a new focus on the prevention of ill-health. Policymakers should consider better monitoring and transparency of voluntary targets or making the policy mandatory to speed up the rate of salt reformulation.

### Perspectives

Hypertension and heart disease are leading causes of morbidity and mortality globally—our results support the implementation of salt reduction targets for the food industry as a strategy to reduce population sodium intake and prevent IHD. Future research should focus on assessing the current salt content of processed foods and the industry’s adherence to the targets, as well as understanding the differences in compliance between voluntary and mandatory programmes.

## ARTICLE INFORMATION

### Author Contributions

L. Bandy, M. Luick, and B. Amies-Cull had full access to all of the study data and took full responsibility for the integrity and accuracy of the analysis. L. Bandy and P. Scarborough are responsible for the conceptualization of the study. L. Bandy, B. Amies-Cull, M. Luick, L.J. Cobiac, and P. Scarborough contributed to the study design and analysis. L. Bandy and B. Amies-Cull wrote the first draft, with contributions from all other authors. All authors reviewed and approved the final article.

### Sources of Funding

L. Bandy, M. Luick, and S.A. Jebb were funded by the National Institute for Health and Care Research (NIHR) Applied Research Collaboration (ARC) for Oxford and Thames Valley. S.A. Jebb was also funded by the NIHR Oxford Biomedical Research Centre (BRC). P. Scarborough and B. Amies-Cull were supported by the NIHR Oxford Health BRC. L.J. Cobiac is supported by a National Health and Medical Research Council Center of Research Excellence grant (No. 2006620).

### Disclosures

None.

### Supplemental Material

Title page

Supplementary References

Tables S1–S8

Figures S1–S6

## Supplementary Material
