# Supplementary material for: Estimating the Potential Impact of the 2024 UK Salt Reduction Targets on Cardiovascular Health Outcomes and Health Care Costs in Adults: A Modeling Study
Source: Hypertension. 2026 Jan 26;83(3):e25159. doi: 10.1161/HYPERTENSIONAHA.125.25159 (PMC12928786; doi:10.1161/HYPERTENSIONAHA.125.25159)
Supplement: Supplementary file 1 [file hyp-83-e25159-s001.pdf]

Supplementary file

Estimating the potential impact of the 2024 UK salt reduction targets on cardiovascular health outcomes and healthcare costs in adults: A modelling study

Lauren Bandy[1], Ben Amies-Cull[1], Madison Luick[2], Linda J. Cobiac[3], Susan A. Jebb[1] and Peter Scarborough[1]

[1]Nuffield Department of Primary Care Health Sciences, University of Oxford, Radcliffe Primary Care Building, Woodstock Road, Oxford, OX2 6GG, United Kingdom

[2]Oxford Population Health, Big Data Institute Building, Old Road Campus, Roosevelt Drive, Oxford, OX3 7LF, United Kingdom

[3]School of Medicine and Dentistry, Griffith University, Kessels Road, Brisbane, Queensland, Australia

Supplementary file order:

Supplementary References

Table S1: UK salt reduction targets 2024 with numbers matched to NDNS foods

Table S2: PRIMEtime input Relative Risks

Table S3: PRIMEtime input baseline utility values and disease utility decrements

Table S4: PRIMEtime input healthcare costs

Table S6: Results of sensitivity analyses 1 and 2

Table S7: Results of sensitivity analysis 3 – daily salt intake

Table S8: Results of sensitivity analysis 3 - estimated impact on quality adjusted life years (QALYs), disease burden, and healthcare costs

Table S5: Population numbers

Figure S1: Baseline disease incidence

Figure S2: Baseline disease prevalence

Figure S3: Baseline disease case fatality rates

Figure S4: Disease incidence trends

Figure S5: Disease case fatality trends

Figure S6: Baseline all-cause mortality rates

Supplementary References

Abbafati, C., Abbas, K. M., Abbasi-Kangevari, M., et al (2020). Global burden of 87 risk factors in 204 countries and territories, 1990–2019: a systematic analysis for the Global Burden of Disease Study 2019. The Lancet, 396(10258), 1223–1249. https://doi.org/10.1016/S0140-6736(20)30752-2

Barendregt, J. J., van Oortmarssen, G. J., Vos, T., & Murray, C. J. L. (2003). A generic model for the assessment of disease epidemiology: The computational basis of DisMod II. Population Health Metrics, 1(1), 1–8. https://doi.org/10.1186/1478-79541-4/TABLES/3

Briggs, A. D. M., Scarborough, P., & Wolstenholme, J. (2018). Estimating comparable English healthcare costs for multiple diseases and unrelated future costs for use in health and public health economic modelling. PLoS ONE, 13(5). https://doi.org/10.1371/journal.pone.0197257

Cobiac, L. J., Law, C., & Scarborough, P. (2022). PRIMEtime: an epidemiological model for informing diet and obesity policy. MedRxiv, 2022.05.18.22275284. https://doi.org/10.1101/2022.05.18.22275284

Lewington, S., Clarke, R., Qizilbash, N., Peto, R., & Collins, R. (2002). Age-specific relevance of usual blood pressure to vascular mortality: A meta-analysis of individual data for one million adults in 61 prospective studies. Lancet, 360(9349), 1903–1913. https://doi.org/10.1016/S0140-6736(02)11911-8

Office for National Statistics. (2021). Population estimates for the UK, England and Wales, Scotland and Northern Ireland: mid-2020. https://www.ons.gov.uk/peoplepopulationandcommunity/populationandmigration/populationestimates/bulletins/annualmidyearpopulationestimates/mid2020

Sullivan, P. W., Slejko, J. F., Sculpher, M. J., & Ghushchyan, V. (2011). Catalogue of EQ5D Scores for the United Kingdom. Http://Dx.Doi.Org/10.1177/0272989X11401031, 31(6), 800–804. https://doi.org/10.1177/0272989X11401031

Table S1: UK salt reduction targets 2024 with numbers matched to NDNS foods

Retail or Out of Home target	Target category code	Salt target category name	Average salt content target (g salt per 100g)	Target matched to NDNS	Number of unique NDNS foods matched
Retail	1.1	Bacon	2.59	Y	21
Retail	1.2	Ham	1.63	Y	16
Retail	1.3.1	Sausages (fresh)	1.08	Y	23
Retail	1.3.2	Sausages (cooked)	1.30	Y	1
Retail	1.4.1	Deli, pork pies and sasage rolls	0.93	Y	12
Retail	1.4.2	Cornish and meat-based pasties	0.80	Y	2
Retail	1.4.3	Other meat-based pastry products	0.60	Y	18
Retail	1.5.1	Cooked and uncured meat - fresh whole muscle	0.68	Y	5
Retail	1.5.2	Reformed whole muscle	0.90	Y	50
Retail	1.5.3	Comminuted or chopped reformed meat	1.35	Y	54
Retail	1.6	Burgers and grill steaks	0.68	Y	36
Retail	1.7.1	Canned frankfurters, hotdogs and burgers	1.30	Y	1
Retail	1.7.2	Fresh chilled frankfurters	1.50	Y	1
Retail	2.1	Bread and rolls	0.85	Y	78
Retail	2.2	Bread and rolls with additions	0.90	Y	1
Retail	2.3	Morning goods - yeast raised	0.65	Y	30
Retail	2.4	Morning goods - powder raised	1.01	Y	15
Retail	3.1	All breakfast cereals	0.48	Y	107
Retail	4.1	Cheddar and other hard-pressed cheeses	1.66	Y	21
Retail	4.2.1	Soft white cheese	0.50	Y	13
Retail	4.2.2	Cottage cheese	0.45	Y	3
Retail	4.3	Mozzarella	1.35	N	0
Retail	4.4	Blue cheese	1.80	Y	3
Retail	4.5.1	Processed cheese spreads	1.55	Y	7
Retail	4.5.2	Other processed cheese	1.63	Y	17
Retail	5.1	Salted butter and buttery spreads	1.33	Y	4
Retail	5.2	Lightly salted butter and buttery spreads	1.06	N	0
Retail	6.1	Margarines and other spreads	0.95	Y	30
Retail	7.1	Baked beans in tomato sauce without accompaniments	0.56	Y	1
Retail	7.2	Baked beans and canned pasta with accompaniments	0.60	Y	1
Retail	8.1	Ready meals and meal centres	0.60	Y	172
Retail	8.2	Ready meals sides	0.69	Y	20
Retail	9.1	Soups (all soups including dried soups as consumed)	0.50	Y	48
Retail	10.1	Pizzas with cured meat toppings	1.00	Y	2
Retail	10.2	Pizzas with other toppings	0.90	Y	9
Retail	11.1	Standard potato crisps	1.25	Y	6
Retail	11.2	Extruded and sheeted snacks	1.61	Y	15

Retail	11.3	Pelleted snacks	2.03	Y	12
Retail	11.4	Salt and vinegar products	1.78	N	0
Retail	11.5	Savoury popcorn	1.23	Y	3
Retail	11.6	Sweet popcorn	0.76	N	0
Retail	11.7	Flavoured nuts	1.00	Y	8
Retail	12.1	Cakes	0.40	Y	81
Retail	12.2	Pastries	0.33	Y	4
Retail	12.3	Sweet pies and other shortcrust or choux pastry-based desserts	0.23	Y	32
Retail	13.1	Bought sandwiches with high salt fillings	0.85	N	0
Retail	13.2	Bought sandwiches without high salt fillings	0.64	N	0
Retail	14.1	Tomato ketchup	1.63	Y	2
Retail	14.2	Brown sauce	1.20	Y	1
Retail	14.3	Salad cream	1.50	N	0
Retail	14.4.1	Mayonnaise (not reduced fat/calorie)	1.25	Y	3
Retail	14.4.2	Mayonnaise (reduced fat/calorie)	1.61	Y	2
Retail	14.5	Salad dressing	1.50	Y	9
Retail	14.6	Chilli sauce	2.88	Y	2
Retail	14.7	Dips	0.75	N	0
Retail	14.8	All other condiments	1.80	Y	32
Retail	15.1	All cook in and pasta sauces (exluding pesto and other thick sauces)	0.68	Y	34
Retail	15.2	Pesto and other thick sauces	1.30	Y	2
Retail	15.3	Thick pastes	3.09	Y	1
Retail	16.1	Sweet biscuits	0.55	Y	94
Retail	16.2	Savoury biscuits	1.30	Y	27
Retail	17.1	Pasta and noodles, plain and flavoured	0.43	Y	37
Retail	18.1	Unflavoured rice	0.15	Y	23
Retail	18.2	Flavoured rice as consumed	0.43	Y	3
Retail	19.1	Other cereals including ready made pastries, yorkshire puddings, batters and mixes	0.53	Y	12
Retail	20.1	Dessert mixes	0.45	Y	5
Retail	20.2	Cheesecake	0.26	Y	6
Retail	20.3	Sponge-based puddings	0.38	Y	11
Retail	20.4	All other processed puddings	0.16	Y	33
Retail	21.1	All quiches	0.50	Y	3
Retail	22.1	All scotch eggs	0.78	Y	2
Retail	23.1	Canned tuna	0.85	Y	8
Retail	23.2	Canned salmon	0.80	Y	6
Retail	23.3	Other canned fish	0.81	Y	14
Retail	24.1	Canned and bottled vegetables	0.13	Y	42

Retail	24.2	Canned and processed marrowfat and mushy peas	0.45	Y	4
Retail	25.1	Plain meat alternatives	0.63	Y	4
Retail	25.2	Meat-free products (including burgers and sausages, pies)	0.85	Y	26
Retail	25.3	Meat-free bacon	1.78	Y	1
Retail	26.1	Dehydrated and instant potatoes	0.15	Y	2
Retail	26.2	Other processed potato products (including frozen chips, wedges and waffles)	0.39	Y	27
Retail	27.1	Dried beverages (including hot chocolate and instant coffee and malted drinks	0.14	Y	26
Retail	28.1	Stocks, as consumed	0.71	Y	1
Retail	28.2	Gravies, as consumed	0.85	Y	11
Out of Home	1.1	Seasoned fries	0.88	Y	1
Out of Home	1.2	Other seasoned potato products	1.35	N	0
Out of Home	2.1	Small burgers without cheese or cured meats	2.15	Y	5
Out of Home	2.2	Burgers with cured meats	4.00	Y	2
Out of Home	2.3	All other burgers	3.33	N	0
Out of Home	3.1	Battered or breaded chicken or portions under 200kcal	0.90	Y	2
Out of Home	3.2	Battered or breaded chicken or portions under 200-400kcal	1.80	N	0
Out of Home	3.3	Battered or breaded chicken or portions over 400kcal	3.15	N	0
Out of Home	4.1	Fish fillet meals	2.75	N	0
Out of Home	4.2	Bite size seafood meals	3.56	N	0
Out of Home	5.1	Pie based meals	3.83	N	0
Out of Home	5.2	Pies only	1.80	N	0
Out of Home	6.1	Curry main meals	3.80	Y	13
Out of Home	6.2	All other sauce based main meals	3.20	Y	7
Out of Home	7.1	Beef steaks, grilled chicken and roast meat main meals	4.05	Y	1
Out of Home	8.1	Cured meat sandwiches	3.38	Y	2
Out of Home	8.2	All other sandwiches	2.48	N	0
Out of Home	9.1	Lasagne, risotto, gnocci and pasta with cured meat	3.56	N	0
Out of Home	9.2	All other pasta dishes	2.75	N	0
Out of Home	10.1	Takeaway style pizza with cured meat toppings	1.13	N	0
Out of Home	10.2	Takeaway style pizza with other toppings	0.88	N	0
Out of Home	10.3	Traditional Italian style pizza with cured meat toppings	6.00	N	0

Out of Home	10.4	Traditional Italian style pizza with other toppings	4.75	N	0
Out of Home	11.1	All children's main meals	1.71	N	0
				**TOTAL**	**1532**

Supplementary file: Model inputs

Table S2: PRIMEtime input Relative Risks

**Disease**	Per (mmHg)	Age group (years)	Mean	Standard deviation	References
IHD	20	<40	0.49	0.04	(Lewington et al., 2002)
IHD	20	40-49	0.49	0.04	
IHD	20	50-59	0.50	0.02	
IHD	20	60-69	0.54	0.01	
IHD	20	70-79	0.60	0.01	
IHD	20	80-89	0.67	0.02	
IHD	20	90+	0.67	0.02	
Stroke	20	<40	0.36	0.06	(Lewington et al., 2002)
Stroke	20	40-49	0.36	0.06	
Stroke	20	50-59	0.38	0.03	
Stroke	20	60-69	0.43	0.02	
Stroke	20	70-79	0.50	0.02	
Stroke	20	80-89	0.67	0.03	
Stroke	20	90+	0.67	0.03	

Table S3: PRIMEtime input baseline utility values and disease utility decrements

**State**	Mean	Standard Deviation	
Male	0.0010046	0.0006241	(Sullivan et al., 2011)
Age 10-19	0.913	0.0045	
Age 20-29	0.905	0.0021	
Age 30-39	0.879	0.0021	
Age 40-49	0.837	0.0028	
Age 50-59	0.798	0.0035	
Age 60-69	0.774	0.0039	
Age 70-79	0.723	0.0049	
Age 80-89	0.657	0.0075	
IHD (incident)	-0.0625727	0.024688167	
IHD (prevalent)	-0.0367975	0.015476334	
Stroke (incident)	-0.1170501	0.018794485	
Stroke (prevalent)	-0.0731964	0.031490105	

Table S4: PRIMEtime input healthcare costs

**HEALTH CARE COSTS**	Mean	Standard Deviation	References
IHD	£606.32	121.26	(Briggs et al., 2018;
Stroke	£2,085.87	417.17	Cobiac et al., 2022)

Table S5: Population numbers

Age group	Male	Female	References
0	307,173	292,188	(Office for National Statistics, 2021)
1-4	1,302,977	1,238,810	
5-9	1,729,978	1,646,887	
10-14	1,722,800	1,639,417	
15-19	1,623,474	1,546,208	
20-24	1,723,088	1,715,218	
25-29	1,826,735	1,914,092	
30-34	1,892,243	2,022,378	
35-39	1,847,714	1,945,068	
40-44	1,735,198	1,791,101	
45-49	1,816,552	1,864,916	
50-54	1,923,854	1,982,778	
55-59	1,850,655	1,908,998	
60-64	1,566,574	1,616,677	
65-69	1,335,722	1,419,764	
70-74	1,326,285	1,454,915	
75-79	924,903	1,066,212	
80-84	631,108	802,385	
85-89	348,922	520,453	
90-94	132,190	255,560	
95-100	28,510	85,290	

Table S6: Results of sensitivity analyses 1 and 2 for 20% and 40% loss of effect of the intervention and for variations on discount rate to 3.5%, 1.5% and 0% for health and cost outcomes.

		**Sensitivity Analysis 1: Effect reduction**		**Sensitivity Analysis 2: Discount rates**	
		20%	40%	1.5%	0%
Salt reduction (g/day, 95% CI)	Females	0.75 (0.68-0.81)	0.56 (0.51-0.61)	*(As primary analysis)*	
	Males	1.07 (0.97-1.18)	0.81 (0.73-0.88)	*(As primary analysis)*	
QALYs saved over 100 years (point estimate and 95% UI, thousands)	Females	62 (22.8 – 99.5)	46 (19.4 – 73.3)	155 (62.9 – 226)	285 (110 – 450)
	Males	133 (47.4 – 215)	101 (39.7 – 159)	329 (135 – 478)	591 (224 – 920)
Healthcare costs saved over 100 years (point estimate and 95% UI, £millions)	Females	260 (85.6 – 467)	196 (67.5 - 343	578 (£203 – 1,000	957 (314 – 1,780)
	Males	549 (188 – 938)	414 (148 – 716)	1,200 (424 – 2,040)	1,941 (548 – 3,320)

Table S7: Results of sensitivity analysis 3 – daily salt intake for adults with 95%CI with no adjustment for underreporting of salt intake

	**Female Population**	**Male Population**	**Population Average**
Daily salt intake at baseline, g/day	4.61 (4.41 – 4.81)	5.91 (5.57 – 6.24)	5.21 (5.02 – 5.41)
Daily salt intake after adjustment for salt reduction targets, g/day	3.79 (3.59 – 3.99)	4.77 (4.49 – 5.04)	4.24 (4.08 – 4.41)
Salt reduction, g/day (95% CI)	0.82 (0.76 – 0.87)	1.14 (1.03 – 1.25)	0.97 (0.90 – 1.04)
Salt reduction as proportion of baseline salt intake (%, 95% CI)	16.8 (15.8 – 17.7)	18.3 (17.1 – 19.6)	17.5 (16.7 – 18.3)

Table S8: Results of sensitivity analysis 3 - estimated impact of 2024 salt reformulation targets being met on quality adjusted life years (QALYs), disease burden, and healthcare costs – with no adjustment for underreporting of salt intake

**Outcome**	**Female Population**	**Male Population**	**Population Total**
NHS Health Care Costs (in million £)	-290 (-541, -102)	-592 (-1095, 208)	-882 (-1627, -312)
QALY	69,000 (27,000, 114,000)	146,000 (57,000, 242,000)	215,000 (83,0006 354,000)
Ischemic Heart Disease cases	-24,000 (-39,000, -9,000)	-68,000 (-111,000, -27,000)	-91,000 (-149,000, -36,0002
Ischemic Stroke incident cases	-10,000 (-16,000, -4,000)	-12,000 (-20,000, -5,000)	-22,000 (-36,000, -9,000)

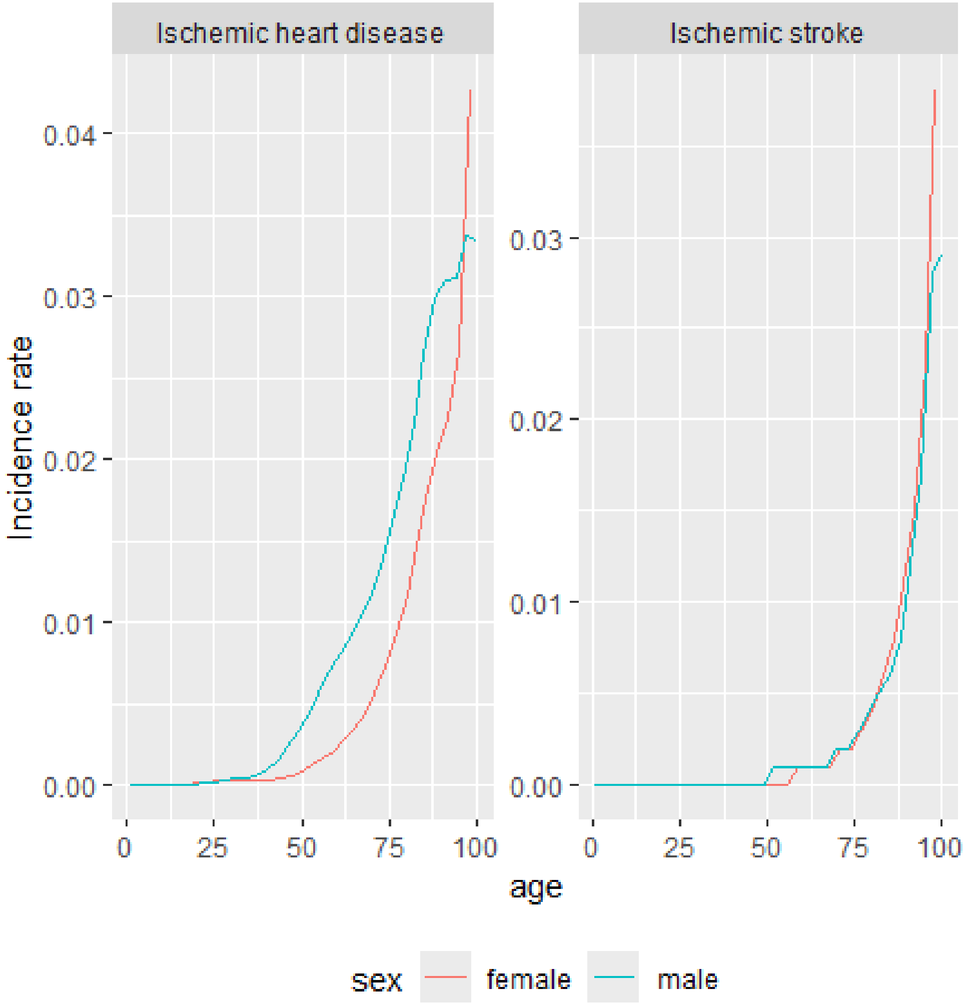

Figure S1: Baseline disease incidence (Abbafati et al., 2020; Barendregt et al., 2003)

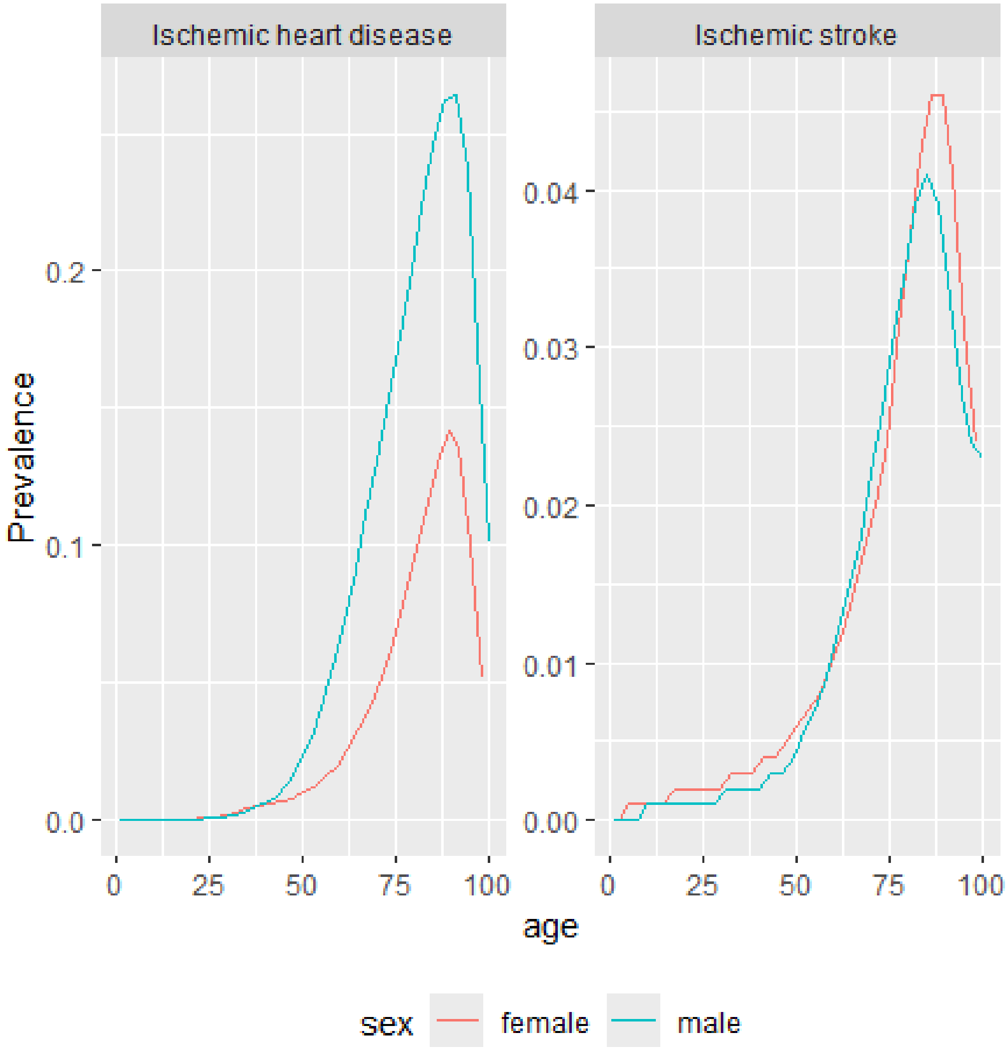

Figure S2: Baseline disease prevalence (Abbafati et al., 2020; Barendregt et al., 2003)

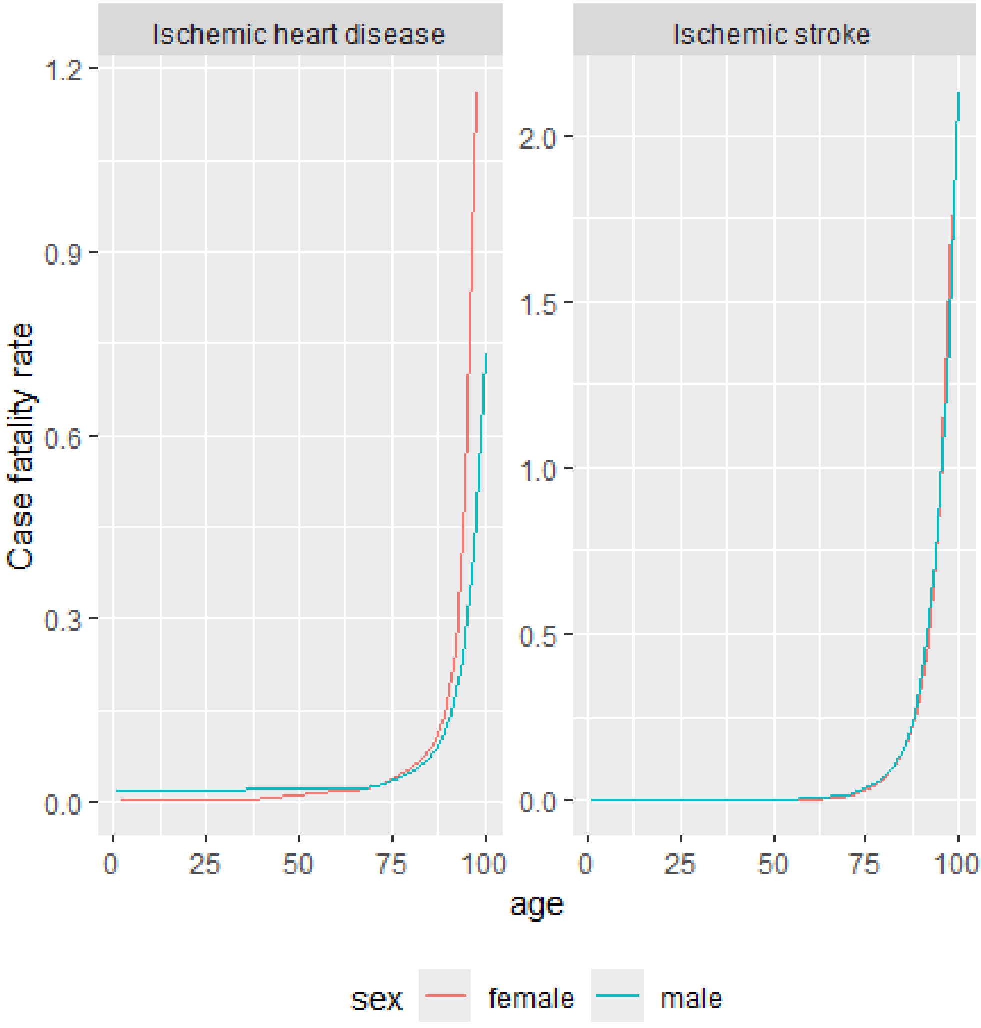

Figure S3: Baseline disease case fatality rates (Abbafati et al., 2020; Barendregt et al., 2003)

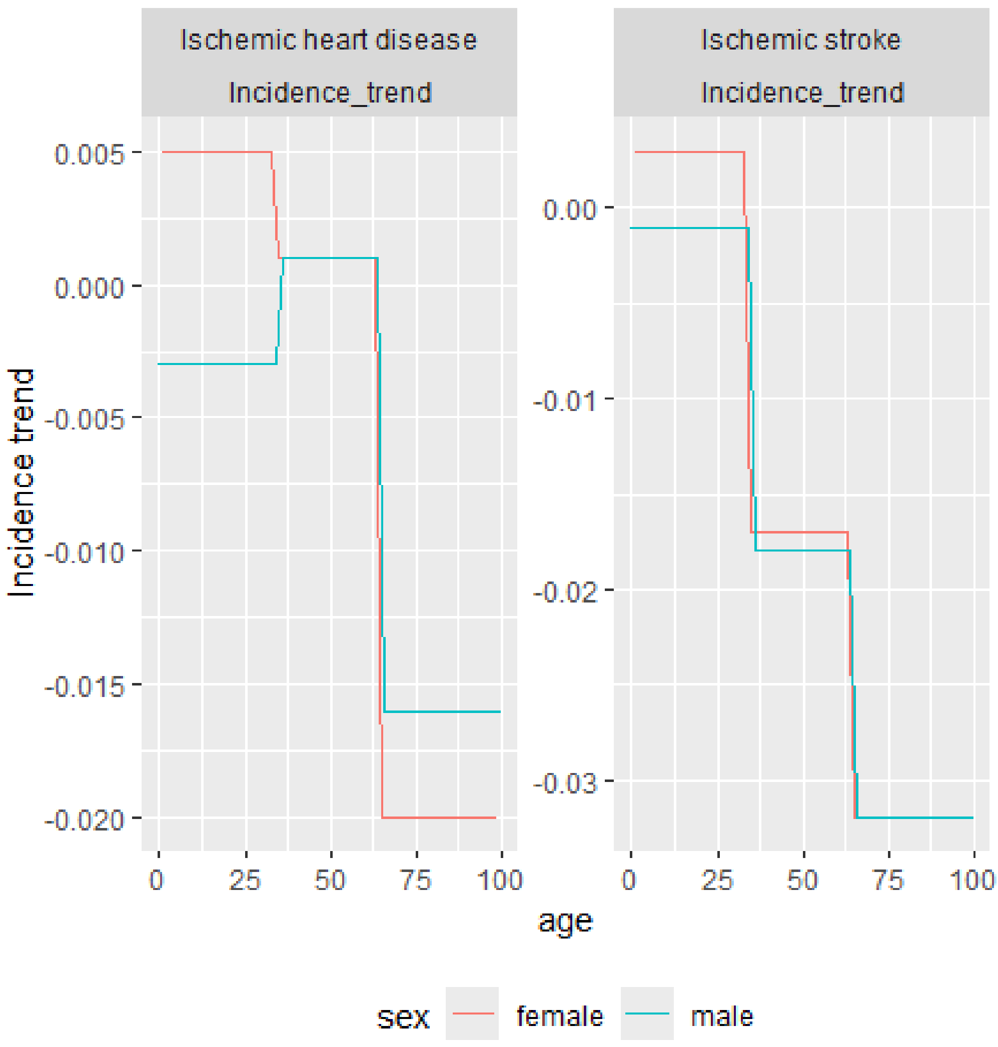

Figure S4: Disease incidence trends (Abbafati et al., 2020; Barendregt et al., 2003)

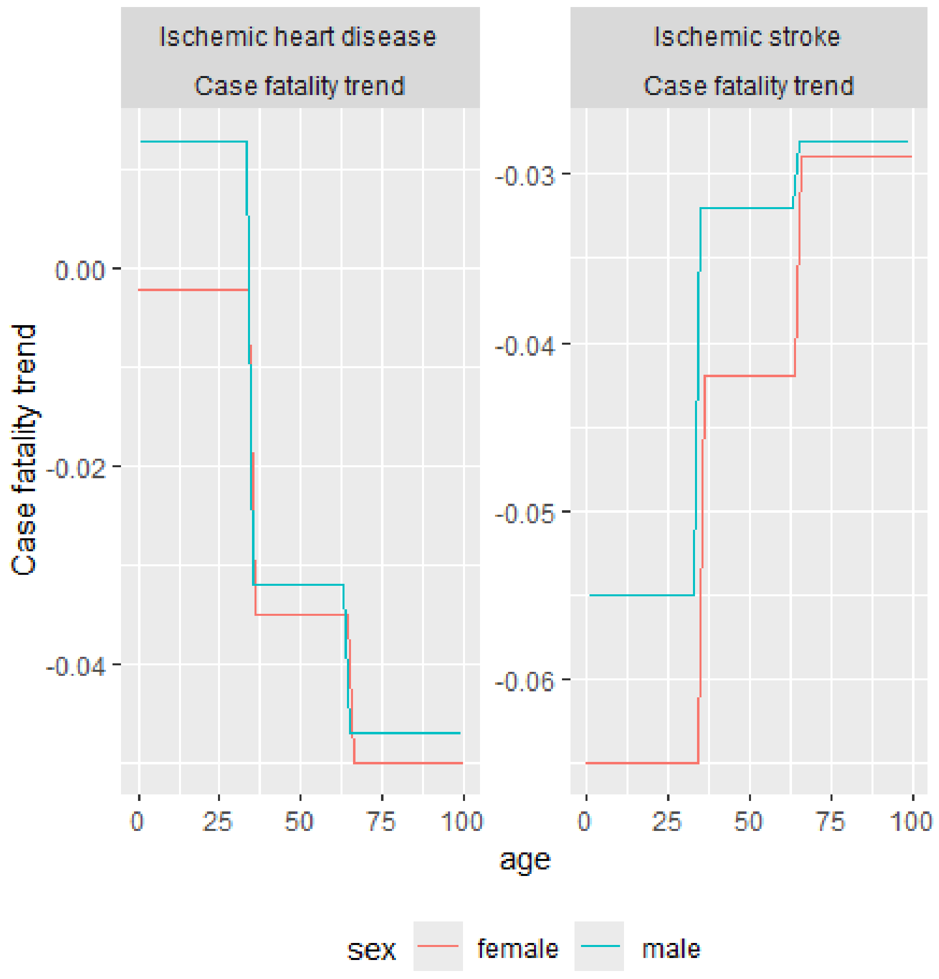

Figure S5: Disease case fatality trends (Abbafati et al., 2020; Barendregt et al., 2003)

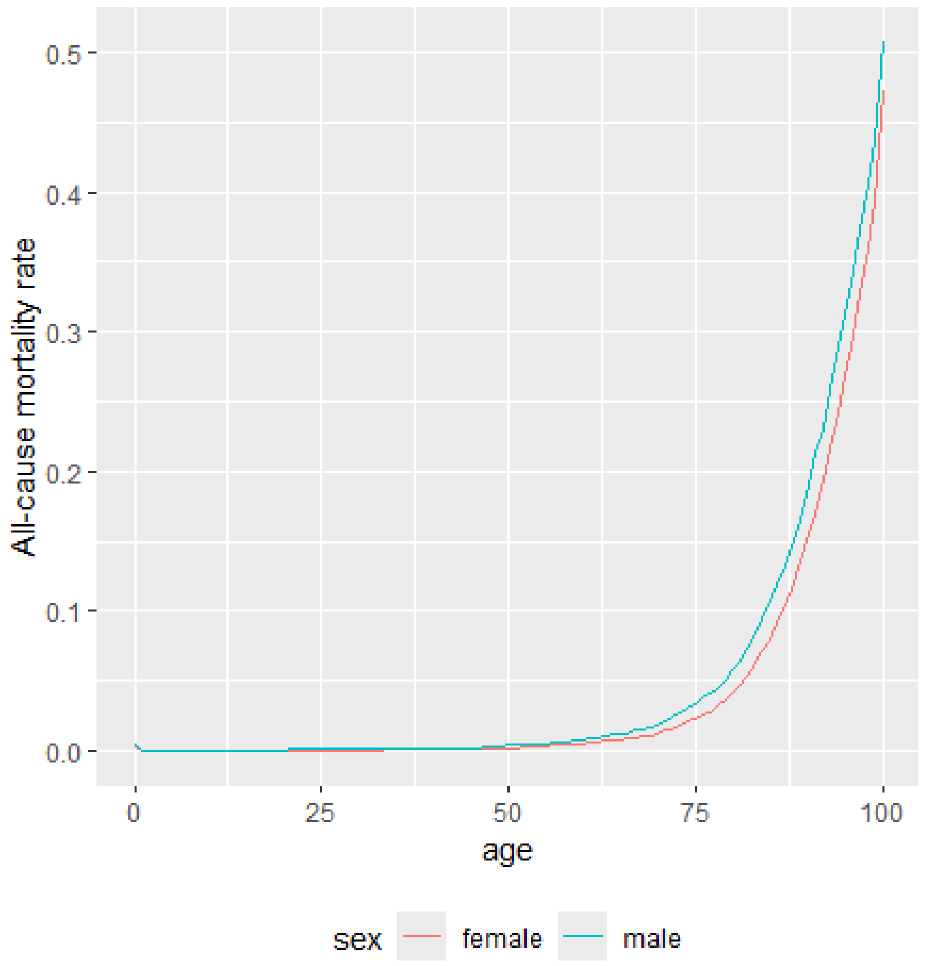

Figure S6: Baseline all-cause mortality rates (Abbafati et al., 2020; Barendregt et al., 2003)
